# Correlations between Functional Imaging Markers Derived from PET/CT and Diffusion-Weighted MRI in Diffuse Large B-Cell Lymphoma and Follicular Lymphoma

**DOI:** 10.1371/journal.pone.0084999

**Published:** 2014-01-15

**Authors:** Xingchen Wu, Hannu Pertovaara, Pasi Korkola, Prasun Dastidar, Ritva Järvenpää, Hannu Eskola, Pirkko-Liisa Kellokumpu-Lehtinen

**Affiliations:** 1 Department of Oncology, Tampere University Hospital, Tampere, Finland; 2 Medical Imaging Centre, Department of Radiology, Tampere University Hospital, Tampere, Finland; 3 Medical School, University of Tampere, Tampere, Finland; 4 Medical Imaging Centre, Department of Nuclear Medicine, Tampere University Hospital, Tampere, Finland; 5 Department of Electronics and Telecommunications, Tampere University of Technology, Tampere, Finland; National Institute of Genomic Medicine, Mexico

## Abstract

**Objectives:**

To investigate the correlations between functional imaging markers derived from positron emission tomography/computed tomography (PET/CT) and diffusion-weighted magnetic resonance imaging (DWI) in diffuse large B-cell lymphoma (DLBCL) and follicular lymphoma (FL). Further to compare the usefulness of these tumor markers in differentiating diagnosis of the two common types of Non-Hodgkin's lymphoma (NHL).

**Materials and Methods:**

Thirty-four consecutive pre-therapy adult patients with proven NHL (23 DLBCL and 11 FL) underwent PET/CT and MRI examinations and laboratory tests. The maximum standardized uptake value (SUV_max_), metabolic tumor volume (MTV), and metabolic tumor burden (MTB) were determined from the PET/CT images. DWI was performed in addition to conventional MRI sequences using two b values (0 and 800 s/mm^2^). The minimum and mean apparent diffusion coefficient (ADC_min_ and ADC_mean_) were measured on the parametric ADC maps.

**Results:**

The SUV_max_ correlated inversely with the ADC_min_ (r = −0.35, p<0.05). The ADC_min_, ADC_mean_, serum thymidine kinase (TK), Beta 2-microglobulin (B2m), lactate dehydrogenase (LD), and C-reactive protein (CRP) correlated with both whole-body MTV and whole-body MTB (p<0.05 or 0.01). The SUV_max_, TK, LD, and CRP were significantly higher in the DLBCL group than in the FL group. Receiver operating characteristic curve analysis showed that they were reasonable predictors in differentiating DLBCL from FL.

**Conclusions:**

The functional imaging markers determined from PET/CT and DWI are associated, and the SUV_max_ is superior to the ADC_min_ in differentiating DLBCL from FL. All the measured serum markers are associated with functional imaging markers. Serum LD, TK, and CRP are useful in differentiating DLBCL from FL.

## Introduction

Non-Hodgkin's lymphoma (NHL) represents a heterogeneous group of lymphoid malignancies that display varying patterns of biological behavior and response to treatment [1]. Prognosis of patients with NHL is affected by the stage, grade, and histological subtype. The most common subtypes of NHL affecting adults are diffuse large B-cell lymphoma (DLBCL) and follicular lymphoma (FL), which together account for more than 50% of the incidences of the disease [2]. Prognostic tumor markers may help to identify high-risk patients who might benefit from more aggressive therapy. Positron emission tomography/computed tomography (PET/CT) with the use of 2-deoxy-2-[^18^F]fluoro-D-glucose (^18^F-FDG) is an established imaging modality that has been proven to be of benefit in the management of malignant lymphomas [3]. Among the PET parameters, standardized uptake value (SUV) is currently the most commonly used semi-quantitative index of ^18^F-FDG metabolic rate. SUV reflects tumor glucose metabolism, and is commonly represented by the mean (SUV_mean_) or maximum (SUV_max_) value. In PET image analysis, SUV_max_ has the advantage of being relatively operator independent. However, the measurement of SUV_max_ has been confined to detection of the most obvious metabolic activities of the tumor at a single site, but not the overall tumor activity. SUV_mean_ is the average value generated from the entire tumor, but differences in operator contouring of tumor will yield varying values. In addition, both SUV_max_ and SUV_mean_ represent only the metabolic activity per gram of tissue, but they are not able to reflect tumor dimensions and volume. In contrast, metabolic tumor burden (MTB) (i.e., total lesion glycolysis) is a newly proposed tumor marker in which both tumor activity and volume are integrated. MTB is the product of SUV_mean_ and metabolic tumor volume (MTV). Lymphoma patients can have single or multiple lesions depending on the stage of the disease. In order to take the number of lesion into consideration, SUV_sum_ (summation of SUV_max_ for all tumors), whole-body MTV (MTV_wb_; summation of MTV for all tumors) and whole-body MTB (MTB_wb_; summation of MTB for all tumors) were calculated in the present study and used as indexes that could potentially reflect overall tumor activity or malignant process of the entire body [4].

Cancer is not only characterized by pathological metabolism, but also by the higher cellularity and morphological changes of tumor cell and tissue [5]. Therefore, restricted water diffusion has been found to be a common feature of tumors. Recent studies have shown that diffusion-weighted magnetic resonance imaging (DWI) is a valuable imaging modality for detecting metastasis and cancer relapse [6]. DWI with apparent diffusion coefficient (ADC) mapping provides information on tumor tissue aggressiveness. ADC value has been applied to distinguish benign from malignant lymph nodes [7], [8], and it has also been used to assess treatment response in various malignancies including lymphoma [6], [9]. Tumors in NHL are typically heterogeneous and can have different histological grades or subtypes in different tumors of the same patient or even in a single tumor [8], [10]. Various components may influence the mean ADC (ADC_mean_) of the tumor/tumors. In contrast, the minimum ADC (ADC_min_) is the most malignant site within the heterogeneous tumor/tumors [11]. In order to compare the diagnostic significance of DWI, both ADC_min_ and ADC_mean_ were measured in the present study.

Both PET/CT and DWI are established imaging modalities in tumor assessment including tumor aggressiveness, treatment response, and prognosis, but they measure different aspects of tumor pathophysiology. A few studies have recently compared FDG-PET/CT and DWI in patients with different cancers, and an inverse correlation between SUV_max_ and ADC_min_ has been revealed [12]–[16]. There are also several well-known prognostic clinical and laboratory predictors for newly diagnosed malignant lymphoma, such as International Prognostic Index (IPI), Follicular Lymphoma IPI (FLIPI), and elevated levels of serum thymidine kinase (TK), Beta 2-microglobulin (B2m), lactate dehydrogenase (LD) and C-reactive protein (CRP). The aim of this study was to investigate the relationships between functional imaging markers derived from PET/CT and DWI, as well as serum tumor markers in DLBCL and FL. In addition, the study was desired to compare the usefulness of these tumor markers in differentiating diagnosis of the two common types of aggressive and indolent NHL.

## Materials and Methods

### Patients

Patients were enrolled from our prospective study investigating the potential of PET/CT and MRI for early chemotherapy response evaluation in patients with NHL. The inclusion criteria were: at least 18 years old, histologically proven DLBCL or FL, WHO performance scale (Zubrod score) better than 4. The exclusion criteria were: concomitant previous malignant disease, primary central nervous system lymphoma, pregnancy or lactation, psychosis, diabetes, human immunodeficiency virus infection or acquired immunodeficiency syndrome, or other serious medical conditions that would prevent imaging examination. The study was approved by the Ethics Committee of Tampere University Hospital, and all patients gave written informed consent prior to study entry.

All patients underwent anamnestic and physical examination, standard laboratory tests including the measurement of serum tumor markers such as TK, B2m, LD, and CRP, as well as CT scans of the chest, abdomen, and pelvis. In addition, unilateral bone marrow aspiration and trephine biopsy were performed on each patient. Pathological samples were reviewed by our expert hematopathologists and classified according to the WHO/Revised European-American Lymphoma classification of lymphoid neoplasm. An experienced physician selected the target tumor mass of interest (the region containing the largest tumor or the greatest number of >1 cm lymph nodes) for DWI analysis based on clinical presentation and CT examination. Clinical prognostic indexes, such as Ann Arbor stage and IPI/FLIPI were also evaluated.

### FDG-PET/CT image acquisition

All patients underwent an integrated PET/CT (Discovery STE 16, GE Healthcare, Milwaukee, WI, USA) examination. The PET/CT imaging covered a volume from the skull base to the upper thigh, and was acquired 72±16 (Mean ± SD) minutes after intravenous injection of the ^18^F-FDG tracer (369±22 MBq) under fasting conditions (Patients were informed to fast at least 6 hours, which was confirmed by an interview). The acquisition was in the 3-dimensional (3D) mode with a 128×128 matrix and 70 cm field of view (FOV), 3 minutes per bed position. The PET images were reconstructed using the 3D VUE Point reconstruction algorithm (GE Healthcare) with 2 iterations and 28 subsets. The postfilter used was 6.0 mm FWHM. The acquisition parameters of the CT scanner were: tube voltage, 120 kV; tube current automatic exposure control range, 100–440 mA; noise index, 18.5 HU; rotation speed, 35 mm/rot; pitch, 1.75∶1. The CT images were reconstructed to slice thicknesses of 1.25 mm and 5.0 mm. The total examination time for PET/CT was approximately 30 minutes.

### PET/CT image analysis

The PET/CT images were evaluated visually and quantitatively. The SUV_max_, SUV_mean_, and MTV were measured from each site (tumor or group of tumors). For each PET/CT dataset, the tumor with the most intense ^18^F-FDG uptake among all foci was carefully identified, and the SUV_max_ was measured on the fused PET/CT images using the AW Volume Share™ workstation (GE Healthcare) [17].

For each tumor or group of tumors, the MTV was estimated in a 3D manner by selecting volume of interest (VOI) on the axial image, and the size of VOI was manually regulated on the corresponding coronal and sagittal images to include the entire active tumor in the VOI, and an isocontour threshold of 42% of the SUV_max_ was determined between the background and the maximal pixel value. The SUV_max_, SUV_mean_, and MTV in the VOI were computed automatically by the program [17].

### MRI Protocol

MR imaging was acquired using a 3 Tesla MR System (Siemens Trio-Tim, Erlangen, Germany) with the manufacturer's body and spine array coils. Additionally, a neck coil was used for the cervical region examination. The MR imaging consisted of a whole-body examination from the level of the skull base to the floor of the pelvis in the coronal plane using a parallel acquisition technique. High resolution axial images and DW images were acquired from the target tumor/tumors. The MRI protocol included a coronal T1-weighted turbo spin echo (TSE) imaging, a coronal T2-weighted inversion-recovery imaging, an axial T1-weighted 3D volumetric interpolated breath-hold examination (VIBE) with fat suppression once before and once after gadolinium (Gd)-DOTA (0.2 ml/kg Dotarem®) injection, and an axial T2-weighted TSE imaging once with and once without fat suppression. Before contrast administration, DWI was acquired using a single-shot echo-planar sequence with fat suppression in the axial plane with two b values (0 and 800 s/mm^2^). The diffusion-weighting gradients were applied in all three orthogonal directions. The DWI was performed during normal respiration and the MR parameters were different depending on the location of the target tumor/tumors [18]. After acquisition of the DWI data sets, pixel-by-pixel ADC maps were reconstructed automatically for each patient.

### ADC value measurement

The ADC value of the target tumor/tumors was measured directly on the parametric ADC maps. A region of interest (ROI) was manually placed on every slice of the entire tumor/tumors that appeared as areas of low signal intensity. In order to ensure proper positioning of the ROI on the ADC maps, the corresponding T2-weighted images and contrast-enhanced T1-weighted images were reviewed side-by-side. Any necrotic areas were excluded from the analysis. For ADC measurement the open-source software ImageJ (created by Wayne Rasband, freely downloadable at the NIH website: http://rsb.info.nih.gov/ij/) was used, which lists the intensity of each pixel within every ADC slice in a single ROI output file. In this manner, the minimum and mean ADC values were calculated. The ADC_min_ was defined as the lowest ADC value within all of the slices of the target tumor/tumors. Accordingly, the ADC_mean_ was defined as the mean value of all of the target tumor/tumors pixels in all of the slices.

### Statistical analysis

The statistical analyses were performed using SPSS software. Mann-Whitney U test was used to compare functional imaging and serum markers between the DLBCL and FL groups. The Spearman's correlation coefficient was used to evaluate the correlations between the ADC_min_ or ADC_mean_ and SUV_max_, SUV_sum_, MTV_wb_, or MTB_wb_, as well as correlations between imaging markers derived from PET/CT or DWI and stage, IPI categories, or serum tumor markers. P values less than 0.05 were considered significant. Receiver operating characteristic (ROC) curve was used to determine the cut-off values of SUV_max_, TK, LD, and CRP with the use of the best combination of sensitivity and specificity to differentiate between DLBCL and FL.

## Results

### Patient characteristics

Thirty-four pretherapy patients with DLBCL or FL (17 male and 17 female; mean age, 63 years; range, 32 to 86 years) underwent PET/CT and MRI examinations within two days. Twenty-three patients had DLBCL including 3 patients with concomitant DLBCL and FL, and 11 patients had FL: 10 with FL grade II and one with FL grade III. The clinical characteristics of the 34 study participants and their tumors are shown in Table 1.

**Table 1:** Demographic characteristics, tumor pathology, and clinical staging of 34 patients with DLBCL or FL.

Characteristics	DLBCL (n = 23)	FL (n = 11)	Total (n = 34)
Age (years)			
mean	64	60	63
range	32–86	43–77	32–86
gender			
female	9	8	17
male	14	3	17
Histology	23 DLBCL	10 FL II, 1 FL III	34
Ann Arbor stage			
I	1	0	1
II	5	1	6
III	5	7	12
IV	12	3	15
IPI or FLIPI*			
0–1	5	0	5
2	7	5	12
3	8	4	12
4	3	2	5
Target tumor site			
abdomen	10	6	16
neck	8	1	9
upper thigh	3	1	4
thorax	2	1	3
pelvis	0	2	2

IPI (International Prognostic Index) was used for the evaluation of DLBCL. IPI 1: low risk; IPI 2: low-intermediate risk; IPI 3: high-intermediate risk; IPI 4: high risk. * FLIPI (Follicular Lymphoma International Prognostic Index) was used for FL. FLIPI 0–1: low risk; FLIPI 2: intermediate risk; FLIPI≥3: high risk.

### The SUV and ADC value between DLBCL and FL groups

**Figure 1:**
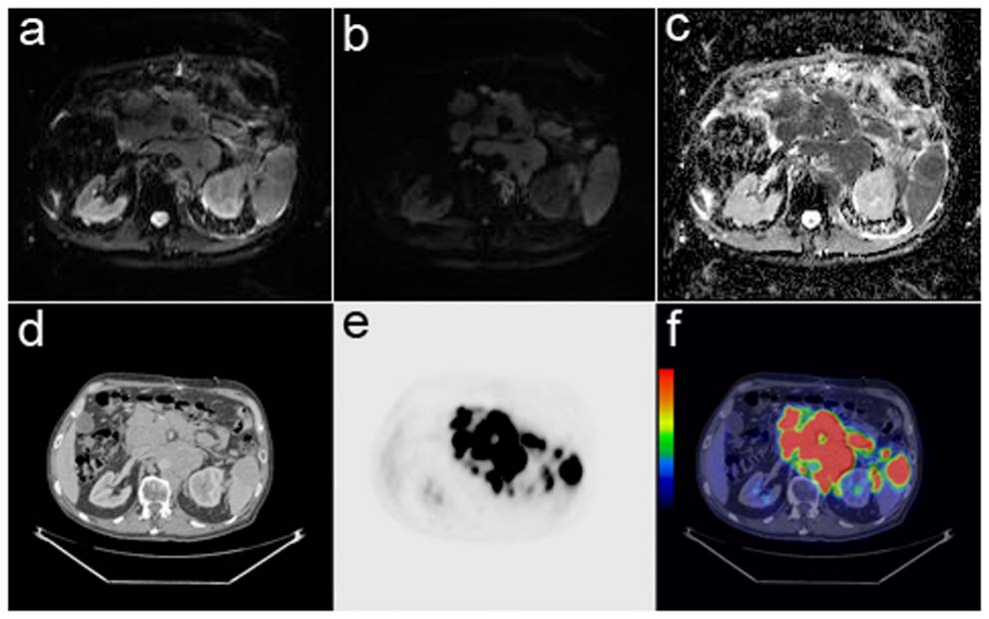
Diffusion-weighted MRI and PET/CT images showing the abdominal region tumor in a 76- year old male patient with diffuse large B-cell lymphoma. (a) B0 image. (b) Diffusion-weighted image with b value 800 s/mm^2^ showed the hyperintensity tumor, but it was not able to depict diffuse spleen involvement. (c) The corresponding ADC map showed the hypointensity tumor with ADC_min_ 0.34×10^−3^ mm^2^/s and ADC_mean_ 0.68×10^−3^ mm^2^/s. (d) Axial CT image. (e) FDG-PET image. (f) The fused PET/CT image showed the active tumor and spleen involvement with SUV_max_ 23.9.

The SUV_max_ was significantly higher in the DLBCL group than that of the FL group (p<0.01) (Table 2 and an example image Figure 1). The only patient with FL grade III had a SUV_max_ of 30.2, which is much higher than the SUV_max_ of those with FL grade II. Serum levels of TK, LD, and CRP were significantly higher in the DLBCL group than in the FL group. However, there was no significant difference in the ADC_min_, ADC_mean_, or serum B2m value between the two groups (Table 2).

**Table 2:** Comparison of PET/CT and DWI indexes and serum biomarkers in the DLBCL and FL groups.

	DLBCL group (N = 23) Median (Mean ± SD)	FL group (N = 11) Median (Mean ± SD)	P value*
SUV_max_	23.9 (21.4±7.6)	10.2 (12.5±7.1)	0.004
SUV_sum_	45.7 (54.0±34.9)	20.0 (48.6±45.0)	0.291
MTV_wb_ (ml)	146 (281±288)	83 (267±423)	0.490
MTB_wb_	1860 (3682±4364)	550 (1658±2215)	0.201
ADC_min_ (×10^−3^ mm^2^/s)	0.40 (0.38±0.11)	0.44 (0.48±0.16)	0.091
ADC_mean_ (×10^−3^ mm^2^/s)	0.76 (0.71±0.17)	0.78 (0.76±0.12)	0.308
TK (U/l)	28.0 (83.0±162.7)	9.9 (14.9±13.7)	0.013
B2m (mg/l)	2.4 (2.8±0.9)	2.1 (2.4±1.0)	0.243
LD (U/l)	292 (449±445)	196 (199±32.7)	0.007
CRP (mg/l)	10.5 (20.0±25.7)	1.4 (13.6±38.3)	0.007

Comparison between the DLBCL and FL groups.

**Figure 2:**
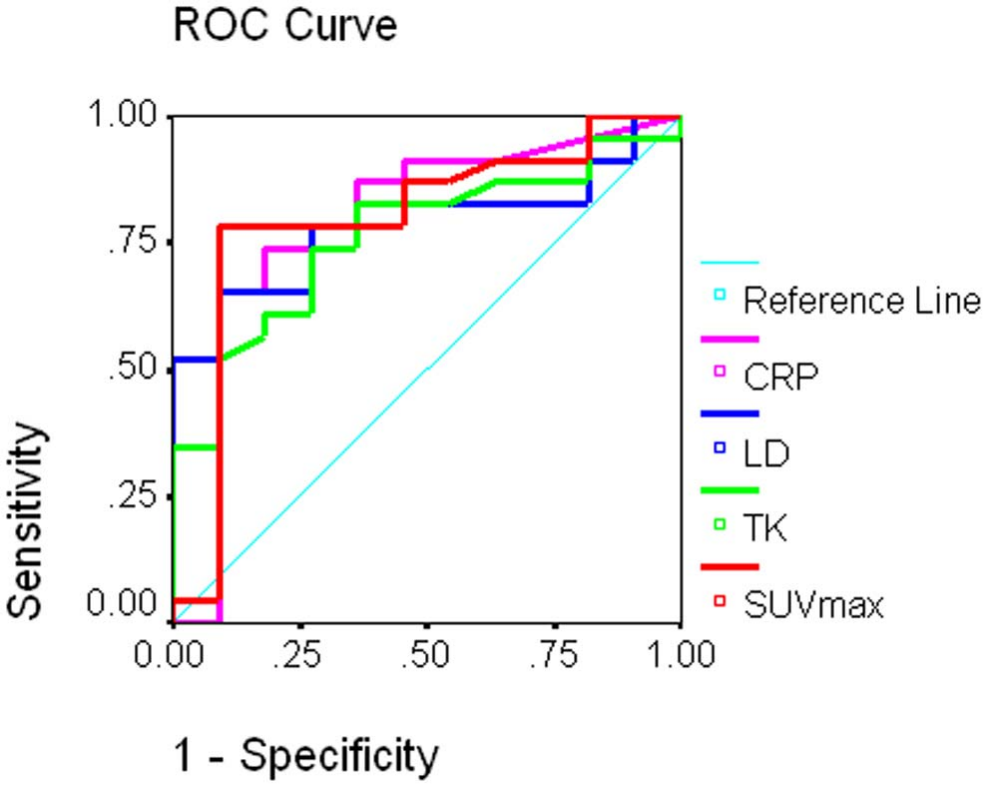
Receiver operating characteristic curve analysis of SUV_max_, serum TK, LD, and CRP in 34 patients with DLBCL or FL. The area under the ROC curve (AUC) was 0.796, 0.763, 0.787, or 0.787 for the SUV_max_, serum TK, LD, or CRP; respectively. When the SUV_max_≥10.5 was used as a cut-off value to differentiate DLBCL from FL, yielded sensitivity 87% and specificity 55%. When a TK cut-off value of ≥10.5 (U/l) or a LD cut-off value of ≥200.5 (U/l) was used to differentiate DLBCL from FL, yielded sensitivity 83% and specificity 64% to detect a DLBCL. When a CRP cut-off value of ≥1.85 (mg/l) was used to differentiate DLBCL from FL, yielded sensitivity 87% and specificity 64% to detect a DLBCL.

To further investigate whether the SUV_max_, serum LD, TK, or CRP can differentiate DLBCL from FL, we used ROC curve analysis (Figure 2). The area under the ROC curve (AUC) was 0.796, 0.763, 0.787, or 0.787; respectively, which suggested that these markers were reasonable predictors for differentiating diagnosis. The SUV_max_ cut-off value of ≥10.5 (normal value <2.5) provided a fair balance, with sensitivity 87% and specificity 55% to detect a DLBCL. When a TK cut-off value of ≥10.5 (U/l) (normal range 0–0.8 U/l) or a LD cut-off value of ≥200.5 (U/l) (normal range 105–205 U/l) was used, yielded sensitivity 83% and specificity 64% to detect a DLBCL. When a CRP cut-off value of ≥1.85 (mg/l) (normal range 0–10 mg/l) was used, yielded sensitivity 87% and specificity 64% to detect a DLBCL. A higher cut-off value would capture more FLs. Conversely, a lower cut-off value would capture a higher percentage of DLBCLs.

### Correlations between SUV and ADC

**Figure 3:**
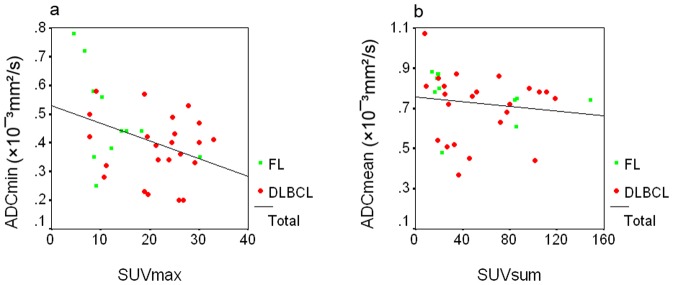
Scatter plots showing the correlations between the SUV_max_ and ADC_min_, (a), and between the SUV_sum_ and ADC_mean_ (b) in 34 patients with DLBCL or FL. The SUV_max_ correlated inversely with the ADC_min_ (r = −0.35, p<0.05) (Figure 3a), and the SUV_sum_ correlated inversely with the ADC_mean_ (r = −0.42, p<0.05) (Figure 3b) in all cases.

The SUV evaluated from PET/CT and the ADC determined from DWI were compared. The SUV_max_ correlated inversely with the ADC_min_ (r = −0.35, p<0.05) in all cases (Figure 3a). No correlation was detected in the DLBCL or FL group when subgroup analysis was performed. The SUV_sum_ correlated inversely with the ADC_mean_ (r = −0.42, p<0.05) in all cases (Figure 3b). The inverse correlation was limited in the FL group (r = −0.73, p<0.05) when subgroup analysis was performed. No correlation was found between the SUV_max_ and ADC_mean_ or SUV_sum_ and ADC_min_.

### Correlations between ADC_min_ or ADC_mean_ and MTV_wb_ or MTB_wb_

**Figure 4:**
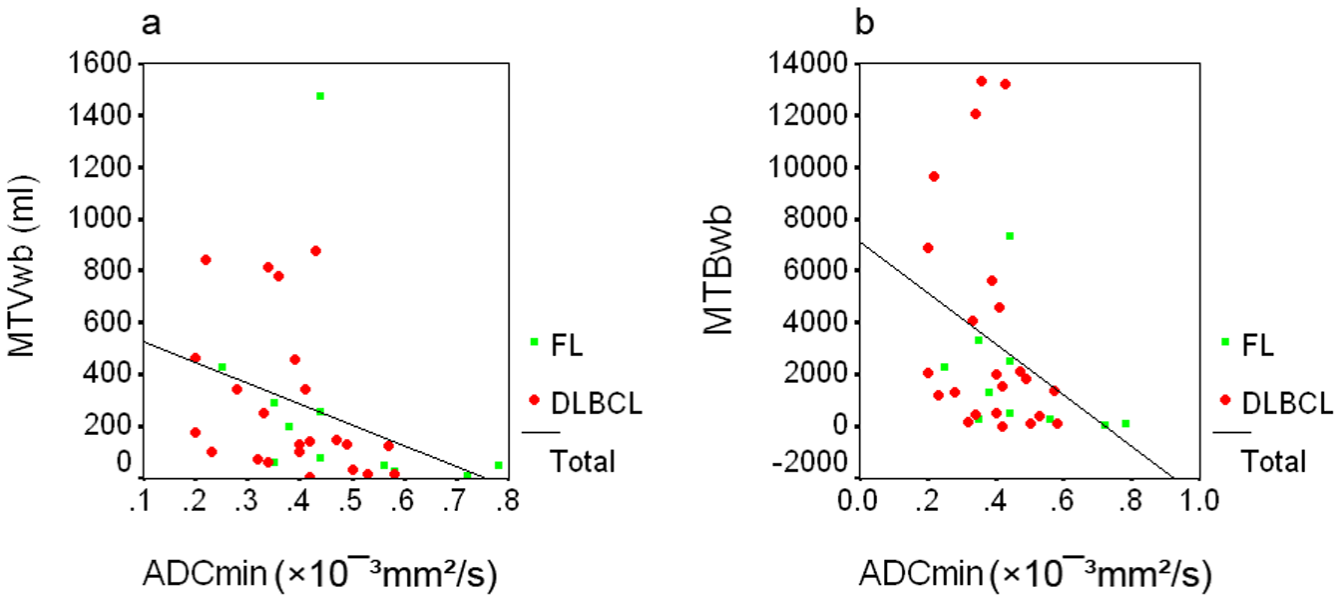
Scatter plots showing the correlations between the ADC_min_ and the MTV_wb_ or MTB_wb_ in 34 patients with DLBCL or FL. The ADC_min_ correlated inversely with the MTV_wb_ (r = −0.54, p<0.01) (Figure 4a), and it also correlated inversely with the MTB_wb_ (r = −0.47, p<0.01) (Figure 4b) in all cases.

The ADC_min_ correlated inversely with MTV_wb_ in all cases (r = −0.54, p<0.01) (Figure 4a). These values also correlated inversely in the DLBCL (r = −0.45, p<0.05) and FL group (r = −0.74, p<0.05); respectively. The ADC_min_ correlated inversely with MTB_wb_ (r = −0.47, p<0.01) in all cases (Figure 4b). The inverse correlation was limited in the FL group (r = −0.69, p<0.05) when the analysis was performed in the subgroups.

**Figure 5:**
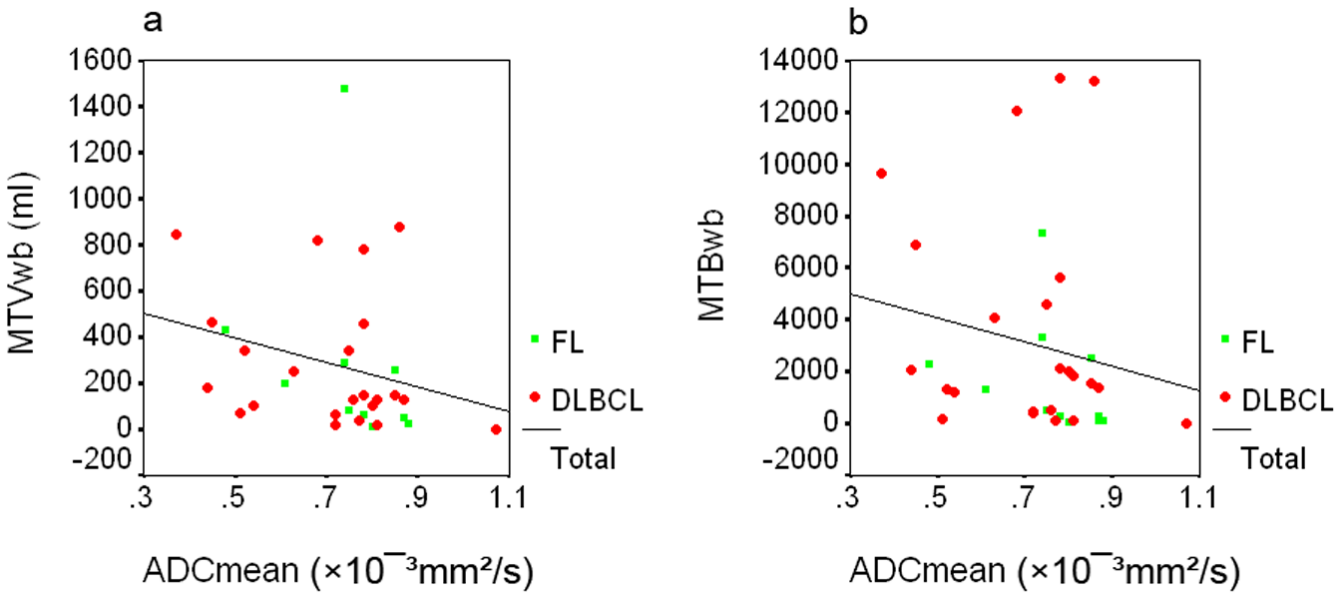
Scatter plots showing the correlations between the ADC_mean_ and the MTV_wb_ or MTB_wb_ in 34 patients with DLBCL or FL. The ADC_mean_ correlated inversely the MTV_wb_ (r = −0.43, p<0.05) (Figure 5a), and it also correlated inversely with the MTB_wb_ (r = −0.35, p<0.05) (Figure 5b) in all cases.

The ADC_mean_ correlated inversely with MTV_wb_ in all cases (r = −0.43, p<0.05) (Figure 5a). The inverse correlation was limited in the FL group (r = −0.75, p<0.01) when the analysis was performed in the subgroups. The ADC_mean_ correlated inversely with MTB_wb_ in all cases (r = −0.35, p<0.05) (Figure 5b). The inverse correlation was limited in the FL group (r = −0.65, p<0.05) when subgroup analysis was performed.

### Correlations between imaging markers and serum markers

The data revealed good correlations between all the measured serum tumor markers (i.e., TK, B2m, LD, and CRP) and MTV_wb_ or MTB_wb_ regardless of disease types (p<0.01; respectively) (Table 3). Subgroup analyses showed that TK, LD, or CRP correlated with both MTV_wb_ and MTB_wb_ (p<0.01; respectively) and B2m correlated with both MTV_wb_ and MTB_wb_ (p<0.05; respectively) in the DLBCL group. TK or B2m correlated with both MTV_wb_ and MTB_wb_ (p<0.05; respectively) in the FL group, but no correlation was detected between LD or CRP and MTV_wb_ or MTB_wb._

**Table 3:** Correlations (Spearman's rho) between clinical stage, IPI, or functional imaging markers and serum markers in 34 patients with DLBCL or FL.

	TK (U/l)	B2m (mg/l)	LD (U/l)	CRP (mg/l)
MTVwb (ml)	r = 0.63 **	r = 0.53 **	r = 0.66 **	r = 0.58 **
MTBwb	r = 0.57 **	r = 0.56 **	r = 0.67 **	r = 0.58 **
SUV_max_	r = 0.31	r = 0.25	r = 0.41 *	r = 0.41 *
ADC_min_ (×10^−3^ mm^2^/s)	r = −0.35 *	r = −0.06.	r = −0.48 *	r = −0.27
Ann Arbor Stage	r = 0.60 **	r = 0.41 *	r = 0.44 *	r = 0.26
IPI or FLIPI	r = 0.28	r = 0.38 *	r = 0.37 *	r = 0.34

p<0.05 and

p<0.01.

In addition, TK correlated inversely with ADC_min_ (r = −0.35, p<0.05), LD correlated with both ADC_min_ (r = −0.48, p<0.01) and SUV_max_ (r = 0.41, p<0.05), and CRP correlated with SUV_max_ (r = 0.41, p<0.05) in all cases (Table 3). Subgroup analysis showed that LD correlated inversely with ADC_min_ in the DLBCL cases (r = −0.48, p<0.05), and LD correlated with SUV_max_ in the FL cases (r = 0.63, p<0.05).

### Correlations between functional imaging markers, serum markers, and IPI categories

There were moderate correlations between the IPI and MTB_wb_ (r = 0.41, p<0.05) or SUV_sum_ (r = 0.42, p<0.05) in the DLBCL group. No correlation was detected between the IPI and ADC value. No correlation was detected between the FLIPI and imaging biomarkers in the FL group. Serum B2m correlated with IPI in the DLBCL group (p<0.05). Serum B2m, LD, or TK correlated with Ann Arbor stage in all cases (p<0.05, respectively) (Table 3).

## Discussion

The IPI, a strong predictor of survival in aggressive NHL, was determined from five factors: age, tumor stage, serum LD concentration, performance status, and number of sites of extranodal involvement. It has been used as the standard clinical tool in the selection of appropriate treatment strategies for individual patients. However, there are still significant differences in outcomes within the same prognostic categories. Therefore, more efficient prognostic markers or models to stratify patients with different survival outcomes are needed. Imaging biomarkers are important for detection and characterization of cancers as well as for monitoring the response to therapy. Integrated PET/CT, with the advantage of combining functional and anatomical information and better attenuation correction, is regarded as current standard reference for the management of lymphomas [3]. Several studies have demonstrated a relation between higher FDG uptake and more aggressive course of malignancy in NHL [19], [20]. High SUV is correlated with rapid cellular proliferation in different subtypes of NHL [19], [21]–[24].

Diffusion-weighted MRI is based on a different approach. In DWI, the random thermal motion of free water molecules known as the Brownian motion can be visualized *in vivo*, and the ADC value varies according to the microstructure and pathophysiological state of the tissues. Within biological tissue, unrestricted free diffusion of molecules does not exist due to cell membranes, organelles and molecular boundaries. In malignant tissue, the microstructural environment that has increased cellularity and dense tumor cell membranes, larger cell nuclei and more abundant macromolecular proteins as well as reduced extracellular space is known to act as a diffusion barrier leading to a decrease of water mobility. On the other hand, necrosis and apoptotic processes may lead to a decrease of cellularity and loss of cell membrane integrity. This, in turn, increases the amount of free water diffusion across the cell membrane and in the extracellular space [6], [8], [15].

In this study, we evaluated the SUV and ADC in patients with DLBCL and FL, and detected an inverse correlation between the SUV_max_ and ADC_min_. This is in agreement with a recent study in Hodgkin's lymphoma (HL) [25]. In addition, several investigators have reported a significant inverse correlation between SUV_max_ and ADC_min_ in various tumors including rectal cancer [13], lung cancer [12], gastrointestinal stromal tumor [14], cervical cancer [15], and endometrial cancer [16]. However, there were also contradictory results. For example, an inverse correlation between the ratio of ADC_min_/ADC_mean_ and SUV_max_/SUV_mean_ was reported in 33 patients with uterine cervical cancer, but neither ADC_min_ nor ADC_mean_ correlated with SUV_max_ or SUV_mean_ [26]. The possible explanation for this finding is that different pathological types of cervical cancer patients including squamous cell carcinoma, adenocarcinoma, and adenosquamous carcinoma were present in the study cohort. In our study, there was a better correlation between the PET/CT and DWI derived markers in the FL group compared with the DLBCL group. This could be explained by the fact that the FL group was relatively homogenous in pathology; all 11 patients had FL grade II, except for one with FL grade III. In contrast, the DLBCL group had obvious pathological variability, including varying differentiating levels of DLBCL and concomitant DLBCL and FL cases. Cancer grows not only with a high proliferation rate, resulting in abnormally high number of cells, but also with architectural alterations in tumor cells and tissue. Changes in nuclear structure are among the most universal of these, a larger cell nuclear size usually means a more aggressive tumor [5]. The decreased ADC value in malignant tumors may be a result of their increased cellularity, larger nuclei with more abundant macromolecular proteins (aggressiveness), and decreased extracellular space. Thus, the ADC value correlates with tumor aggressiveness in specific tumor histological subtype [27]. In contrast, the SUV_max_ reflects the highest tumor metabolic rate, regardless of the underling microstructure changes. Thus, the SUV_max_ and ADC_min_ are independent functional imaging markers that may complement each other in the management of lymphomas, and to use both types of data may improve the accuracy of diagnoses [15].

The SUV_sum_ reflects the total tumor metabolic activity of the whole body and the ADC_mean_ indicates the overall tumor cellularity and aggressiveness. It is not surprising that an inverse correlation between the SUV_sum_ and ADC_mean_ was also detected in this study. To our knowledge, this is the first report of the relation between SUV_sum_ and ADC_mean_, and this finding needs to be verified in future studies. In addition, both the ADC_min_ and the ADC_mean_ correlated inversely with MTV_wb_ and MTB_wb_, which reflect the total metabolic tumor volume or total amount of tumor glycolysis in the patient's body. These correlations could be explained by the fact that a lower ADC value indicates increased aggressiveness. In general, more aggressive tumors proliferate more rapidly and with a higher risk of metastasis, and accordingly the MTV_wb_ and MTB_wb_ are also greater. No correlation was detected between SUV_max_ and ADC_mean_. This is in agreement with our previous lesion-wise comparison of SUV_max_ and ADC_mean_ in DLBCL [10], since the SUV_max_ represents the most malignant site of the heterogeneous tumors, whereas ADC_mean_ indicates the overall tumor aggressiveness.

Both DLBCL and FL had a wide range of FDG avidity. Our result showed that SUV_max_ was a useful marker in differentiating between DLBCL and FL, although overlap between the two types of disease existed. A SUV_max_ cut-off value of ≥10.5 was indicative of aggressive disease. The only patient with FL grade III had much higher SUV_max_ compared with those with FL grade II. This is in agreement with previous studies showing that indolent FL is associated with low-grade FDG uptake, and more intense FDG accumulation has been observed in more aggressive B-cell lymphomas [19], [20]. Although typically biopsies are obtained from areas that are easily accessible, an unexpectedly high SUV_max_ may suggest a transformation of an indolent NHL to a more aggressive disease and a new biopsy of the site with the highest SUV_max_ should be considered. Therefore, our results suggest that PET scanning may also be helpful in directing biopsies and changing clinical management. In contrast, DWI with ADC value measurement is inferior to PET/CT derived SUV_max_ in differentiating DLBCL from FL and is therefore not able to guide biopsy. This finding is concordant with a previous study in a group of 16 indolent and 16 aggressive NHL patients [28]. The possible explanation is that ADC value reflects both tissue cellularity and tumor aggressiveness, but aggressive tumors do not always have higher cellularities. e.g., we have demonstrated that FL had a higher cellularity than DLBCL [17]. Thus far, the most promising oncologic application of DWI seems to be in tumor detection and treatment response evaluation [6].

In our study, serum TK, LD, and CRP were significantly higher in the DLBCL group than in the FL group. ROC analysis showed that they are useful markers in differentiating the two common types of indolent and aggressive lymphoma. Serum LD represents a surrogate quantitative measure of tumor burden and aggressiveness in NHL [29], and it is a one of the components of the IPI. A high serum B2m level is an independent adverse prognostic factor in malignant lymphoma, which is also related to the tumor burden [30], [31]. CRP is an acute-phase reactant, and elevated baseline serum CRP levels have also been found to be a poor prognostic factor in cancers of many types [32], [33]. High serum CRP might reflect a high metastatic potential, as it is known to promote metastatic spread by stimulating angiogenesis, increasing vascular permeability, and acting as an endothelial cell mitogen [34]. Elevated serum TK predicts high proliferation of tumor cells in lymphoma [35]. Our study showed that serum LD, TK, CRP or B2m correlated with both MTV_wb_ and MTB_wb_. Therefore, these serum tumor markers could serve as clinically useful markers in malignant NHL, since they are widely available and relatively inexpensive. In addition, a recent study showed that they are independent prognostic markers [36]. The IPI correlated with quantitative PET/CT functional markers (MTB_wb_ and SUV_sum_) in the DLBCL group. This indicates that the IPI remains a useful and simple clinical tool in evaluating the risk and prognosis of aggressive NHL.

There was a limitation in our study, the PET/CT was a whole body examination, but the DWI was performed only in the target tumor/tumors. As such, ADC_min_ might not be the minimum ADC of the whole body, since the largest tumor was not always the one with the highest malignancy. Additionally, the study included only a small cohort, and future studies with larger patient cohorts are needed to confirm our findings.

### Conclusions

Glucose metabolism with PET/CT and ADC value with DW-MRI are different indexes for the characterization of lymphomas. The functional imaging markers derived from DWI and FDG-PET/CT are associated, and FDG-PET/CT is superior to DWI in differentiating DLBCL from FL. The measured serum tumor markers such as LD, TK, CRP, and B2m are associated with functional imaging markers, and LD, TK, and CRP are useful in differentiating DLBCL from FL.
